# Treatment of retained placenta with misoprostol: a randomised controlled trial in a low-resource setting (Tanzania)

**DOI:** 10.1186/1471-2393-9-48

**Published:** 2009-10-23

**Authors:** Heleen J van Beekhuizen, Andrea B Pembe, Heiner Fauteck, Fred K Lotgering

**Affiliations:** 1 Department of Obstetrics and Gynaecology, Erasmus Medical Centre, PO box 2040, 3000 CA Rotterdam, The Netherlands; 2 Department of Obstetrics and Gynaecology, Muhimbili University of Health and Allied Sciences, Dar es Salaam, Tanzania; 3 Institute for Cancer Epidemiology, University Luebeck, Germany; 4 Department of Obstetrics and Gynaecology, Radboud University Nijmegen Medical Centre, Nijmegen, The Netherlands

## Abstract

**Background:**

Retained placenta is one of the common causes of maternal mortality in developing countries where access to appropriate obstetrical care is limited. Current treatment of retained placenta is manual removal of the placenta under anaesthesia, which can only take place in larger health care facilities. Medical treatment of retained placenta with prostaglandins E1 (misoprostol) could be cost-effective and easy-to-use and could be a life-saving option in many low-resource settings. The aim of this study is to assess the efficacy and safety of sublingually administered misoprostol in women with retained placenta in a low resource setting.

**Methods:**

Design: Multicentered randomised, double-blind, placebo-controlled trial, to be conducted in 5 hospitals in Tanzania, Africa.

Inclusion criteria: Women with retained placenta, at a gestational age of 28 weeks or more and blood loss less than 750 ml, 30 minutes after delivery of the newborn despite active management of third stage of labour.

Trial Entry & Randomisation & Study Medication: After obtaining informed consent, eligible women will be allocated randomly to the treatment groups using numbered envelopes that will be randomized in variable blocks containing identical capsules with either 800 microgram of misoprostol or placebo. The drugs will be given sublingually. The women, maternal care providers and researchers will be blinded to treatment allocation.

Sample Size: 117 women, to show a 40% reduction in manual removals of the placenta (p = 0.05, 80% power). The randomization will be misoprostol: placebo = 2:1

Primary Study Outcome: Expulsion of the placenta without manual removal. Secondary outcome is the number of blood transfusions.

**Discussion:**

This is a protocol for a randomized trial in a low resource setting to assess if medical treatment of women with retained placenta with misoprostol reduces the incidence of manual removal of the placenta.

**Clinical Trial Registration:**

Current Controlled Trials ISRCTN16104753

## Background

### Retained placenta: diagnosis, definition and the burden of disease

The diagnosis 'retained placenta' (RP) is established when the placenta is not expelled after a certain time period following the delivery of the infant[1,2]. The time period in the definition of RP varies among countries. In our study location Tanzania, like most English speaking countries, RP is defined as lack of expulsion of the placenta 30 minutes after delivery of the infant [3], while in other countries the diagnosis RP is only made after 60 minutes postpartum [4]. Complications of RP are postpartum haemorrhage and infection[5], which may both lead to maternal morbidity and mortality. The need to improve maternal mortality has been recognized at a global level by including it in the Millennium Development Goals [6].

Tanzania is a low resource country with a high maternal mortality rate. It is estimated that 578 women per 100,000 live-births die as a result of pregnancy-related complications [7]. A retrospective study in Tanzania showed that RP contributed by 13% to the maternal deaths [8]. The morbidity due to RP is mainly caused by infections and anaemia. A study by Tandberg et al found a significant fall in haemoglobin level postpartum compared to antepartum by a mean of 3.4 g/dl (2.1 mmol/l) in the RP group as compared to no significant change in the controls; blood transfusion was required in 10% of the RP group versus 0.5% in the control group [9].

### Retained Placenta: incidence

The incidence of RP is approximately 1-2% of all deliveries worldwide, the exact data for Tanzania is not known. The reported incidence of RP is affected by the following four factors: definition of the time interval [4], gestational age, an obstetrical history of previous RP or not, and the presence or absence of active management of the third stage of labour (AMTSL).

The incidence of RP in an unselected group of nulliparous women in The Netherlands was 6.3% at 30 minutes and 1.8% at 60 minutes after delivery of the newborn [4]. The incidence of RP, 30 minutes after delivery of the newborn has been reported as 8% in preterm and 1.1% in term deliveries [10]. The recurrence risks of RP reported in two studies of parous women were 16% and 23% [9,11]. A randomized controlled trial (RCT) showed that AMTSL reduced the incidence of RP (after 30 minutes) to 1.6% as compared to 4.6% in the control group [12].

### Retained placenta: how can we prevent maternal mortality?

Blood loss associated with RP can be acute life-threatening and requires emergency interventions like administration of uterotonics, correction of hypovolaemia by administration of intravenous fluids, manual removal of the placenta (MRP) under anaesthesia and blood transfusions[13]. All these interventions need skilled personnel and equipment. In many low-resource countries women deliver at home, and the nearest health care facility often lacks drugs and equipment to perform MRP or to give blood transfusion, leaving the midwife empty-handed. Transport to health care facilities with comprehensive emergency obstetrical care requires time and money, both are limited commodities in those circumstances [6,8]. Medical treatment of RP with an easy-to-administer drug could save the life of patients under those circumstances.

### Retained placenta: medical treatment with prostaglandin analogues

Medical treatment of RP includes the administration of oxytocin in the umbilical vein, which was reported to be effective in one out of eight women (relative risk 0.79, 95% CI 0.69-0.91) [14]. The disadvantage of this method in low resource settings is that it requires skilled medical personnel and equipment.

A RCT in The Netherlands showed that administration of 250 microgram prostaglandin E2 (sulprostone) intravenously 60 minutes after delivery of the infant effectively expelled 49% of RP versus 11% in the placebo group [15] within 60 minutes after administration. Blood loss was 388 ml lower in the sulprostone group (average blood loss 1062 ml) as compared to controls (average blood loss 1450 mls). Unfortunately, treatment with sulprostone is not applicable in low resources settings because the drug is relatively expensive and needs to be stored refrigerated.

The prostaglandin E1 analogue misoprostol is inexpensive and does not need to be stored refrigerated. Therefore, it is of potential use in low-resource countries. In a recent study in which 54 patients with RP were randomised to misoprostol, oxytocin and placebo, administered through the umbilical cord [16], a significant reduction of MRP was reported for the misoprostol (43% MRP) compared to the oxytocin (80% MRP) and placebo (54% MRP) groups. When misoprostol was administered rectally in a group of 10 patients [17], it was reported to avoid MRP in 7 patients and to reduce blood loss. Oral or sublingual administration of misoprostol, though potentially the fastest acting [18] and most practical, has not been studied in an effort to reduce MRP.

### Misoprostol

Misoprostol is an prostaglandin E1-analogue with uterotonic properties that can be administered orally, sublingually, vaginally and rectally [19]. Sublingual administration of misoprostol achieves the highest serum peak concentration and takes the shortest time to reach the peak level, in comparison with other routes of administration [18]. Misoprostol is cheap and stable at room temperature. Originally, misoprostol was introduced as treatment for peptic ulcers. It soon became obvious that it stimulates uterine contractions [20]. Misoprostol has been used to treat various obstetrical problems, including uterine atony, postpartum haemorrhage [21], induction of labour, and induction of abortion [19,20]. Misoprostol when given postpartum is known to cause only mild side effects (shivering and pyrexia) [19,22-24]. Misoprostol is a sustainable drug for use in developing countries for the treatment of various obstetrical complications [20] like postpartum haemorrhage, induction of labour and induction of abortion. It is registered in Tanzania for the prevention and treatment of postpartum haemorrhage.

### Study justification

Women in rural areas in resource-poor settings who deliver at home or in a village health care facility, and in whom the delivery is complicated by RP, have difficulty to reach appropriate medical help in time and have a considerable chance to die because from post partum hemorrhage. Because preliminary evidence suggests that prostaglandins like misoprostol may expel the placenta and reduce blood loss in women with RP, a RCT is designed to compare sublingually administered misoprostol with placebo to tests its effectiveness to reduce the need of MRP and blood transfusion in a low-resource setting.

### Aims of the trial

The aims of this randomised, double blind, placebo-controlled trial is to assess if sublingual misoprostol reduces the need of Manual Removal of Placenta (MRP) and the amount of blood loss in women with RP in a low resource setting. The primary outcome variable is reduction in the incidence of MRP and the secondary outcome variable is the reduction in the number of units of packed cells administered.

The primary hypothesis of this randomised trial is that the administration of misoprostol to women with RP reduces the number of women who need MRP. The secondary hypothesis is that misoprostol reduces the amount of blood loss in women with RP, especially in those in whom the placenta is expelled by the intervention. Since measurement of blood loss during delivery is not always very reliable, we choose as secondary outcome variable both the measured amount of blood loss and the number of administered packed cells.

## Methods/Design

### Study Design

Multicentered randomised, double-blind, placebo-controlled trial.

### Participating hospitals & approval

The study will be conducted in four rural hospitals in Southern Tanzania (the regional hospitals of Lindi and Mtwara regions and Ndanda and Nyangao mission hospitals) and in the university teaching hospital in the capital Dar es Salaam. Approval for this study was obtained from the National Institute of Medical Research (NIMR), the Senate Research and Publication Committee of Muhimbili University of Health and Allied Sciences and the Muhimbili National Hospital in Tanzania. A data management safety board has been installed.

### Inclusion criteria and trial entry

All labouring women will receive AMTSL and are eligible if 30 minutes after delivery of the infant the placenta has not been expelled and were delivered of a baby of 1 kg or more or at a gestational age of 28 weeks or more. AMTSL is defined as administration of 5IU oxytocin and controlled cord traction (CCT). If the placenta is delivered the uterus will be massaged.

### Exclusion criteria

Women with one of the following conditions will be excluded from entering the trial:

• Haemoglobin concentration less than 100 g/l (6.2 mmol/l)

• Blood loss more than 750 ml

• Pulse rate more than 120 beats per minute

• Diastolic blood pressure reduction after delivery more than 20 mmHg

### Trial Entry

**Figure 1:**
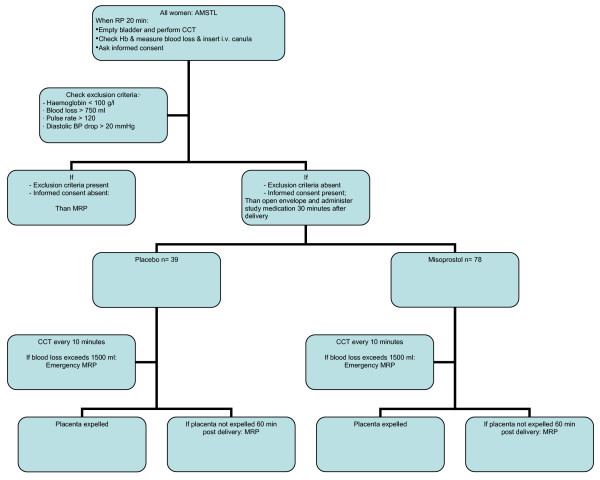
**Flowchart for trial entry**. AMTSL = Active management of third stage of labour. RP = retained placenta. CCT = controlled cord traction. Hb = Haemoglobin. BP = Blood pressure. MRP = Manual removal of placenta. Hb = haemoglobine.

Eligible women will be identified in the labour ward at 20 minutes after delivery of the infant. The bladder will be catheterised, an intravenous canula will be inserted and normal saline solution will be started, CCT will be performed again and blood will be taken for cross-match and haemoglobin concentration. They will receive verbal and written information in Kiswahili about participation in the trial and will be asked to give their informed consent. The randomisation schedule uses balanced variable blocks; sealed envelopes containing both registration form and blinded study medication are present in the delivery room. Allocation will be in sequence of enrolment in each of the five labour wards. Each sealed envelop contains two identical capsules with either 800 microgram misoprostol or placebo. The patient, the maternal care providers and the researchers are all blinded to the allocation. Women will enter the study after giving their informed consent at 30 minutes following the delivery of the infant, at which time the envelope will be opened and the two capsules of study medication will be administered sublingually. Figure 1 depicts the flowchart for trial entry.

### Study Medication & Treatment Schedules

After administration of the study medication, the doctor or midwife will perform CCT every ten minutes to check if the placenta has separated from the uterine wall. Vaginal blood loss will be measured by weighing self absorbable mattresses. Blood loss exceeding 1500 ml will be considered as indication for emergency MRP. If the placenta is not expelled 30 minutes after the administration of the study medication, the patient will undergo MRP.

### Follow-up of women in both treatment groups

All women enrolled in the study are followed up for 12-24 hours. Blood pressure, pulse rate uterine contraction and vaginal blood loss are monitored, and the haemoglobin concentration prior to discharge is recorded. Women are receiving blood transfusion and and/or intravenous iron dextrane infusion, according to the hospitals guidelines, if needed. All women receive combined ferrofumerate and folic acid tablets according to the national guideline on care for post partum women.

### Study Endpoints

The primary study outcome is:

• Manual removal of the placenta.

The secondary outcomes are:

• Measured post partum blood loss,

• Number of units of blood administered,

• Adverse outcome for the woman, including side-effects from the study medication and number of emergency MRP needed.

### Sample Size

The primary endpoint of the study is manual removal of the placenta. For eligible women (with RP 30 minutes after delivery of the infant) the best estimate of MRP is 44% at 60 minutes post partum. Using 2:1 misoprostol to placebo randomisation, a sample size of 117 women will be able to show a 40% reduction in MRP (5% level of significance, two-tailed alpha, 80% power). Thus, 39 patients will receive placebo and 78 will receive misoprostol.

### Analysis and Reporting of Results

Baseline characteristics of all women enrolled in the study are documented and analysed in order to verify the absence of confounding differences in baseline variables between groups. Outcome comparisons for women will be analysed according to 'intention to treat'. Relative risks and 95% confidence intervals will be reported for the primary and secondary outcomes, and the number needed to treat to prevent one MRP will be calculated. A data management safety board will check the data at regular intervals.

## Discussion

This is a protocol for RCT assessing the efficacy of sublingual misoprostol in women with a RP 30 minutes after delivery of the infant. The trial is conducted in a low-resource setting in order to establish if misoprostol treatment of RP reduces maternal morbidity associated with retained placenta in such a setting. This study is partially carried out in an environment where communication is difficult and where people have little experience with conducting research. During the research period we have to anticipate unforeseen difficulties like breakdown of equipment, loss of data or study medication and other unpredictable events.

When the hypotheses of the study, that misoprostol reduces the amount of MRP and blood loss, is confirmed, it should have consequences for the basic obstetrical care in rural health centres in developing countries. Misoprostol should then be made available to all health facilities and staff should be trained in administering the drugs correctly.

## Competing interests

The authors declare that they have no competing interests.

## Authors' contributions

HJvB, ABP, HF and FKL all contributed to the development of the trial protocol. HJvB drafted this manuscript. All authors reviewed the text critically and gave their approval to the final to be published version of the manuscript.

## Pre-publication history

The pre-publication history for this paper can be accessed here:


